# Integrative Analysis of Methylome and Transcriptome Reveals the Importance of Unmethylated CpGs in Non-CpG Island Gene Activation

**DOI:** 10.1155/2013/785731

**Published:** 2013-07-10

**Authors:** Amichai Marx, Tamar Kahan, Itamar Simon

**Affiliations:** Department of Microbiology and Molecular Genetics, IMRIC, The Hebrew University-Hadassah Medical School, 91120 Jerusalem, Israel

## Abstract

*Background*. Promoter methylation is associated with gene repression; however, little is known about its mechanism. It was proposed that the repression of methylated genes is achieved through the recruitment of methyl binding proteins (MBPs) that participate in closing the chromatin. An alternative mechanism suggests that methylation interferes with the binding of either site specific activators or more general activators that bind to the CpG dinucleotide. However, the relative contribution of these two mechanisms to gene repression is not known. *Results*. Bioinformatics analyses of genome-wide transcriptome and methylome data support the latter hypothesis by demonstrating a strong association between transcription and the number of unmethylated CpGs at the promoter of genes lacking CpG islands. *Conclusions*. Our results suggest that methylation represses gene expression mainly by preventing the binding of CpG binding activators.

## 1. Background

It is widely accepted that methylation at CpG residues functions as an important mechanism for maintaining gene repression [1]. Combining methylome and expression profile data allowed a comprehensive analysis of this claim and revealed a strong correlation between methylation of promoters and gene repression [2, 3]. However, the mechanism that links promoter methylation and gene silencing is not yet understood. Two mechanisms by which DNA methylation represses gene expression were identified. First, methylation may interfere with transcription factor binding the DNA by altering their binding sites. This mechanism may explain, for example, the role of methylation in preventing transcription activation by the E2F and CREB transcription factors [4, 5]. A second and a more general mechanism involves the recruitment of methyl-CpG binding proteins which associate with various chromatin modifiers to establish a repressive chromatin structure [6–10]. 

Recent studies have identified a new family of proteins that are recruited to C^Un-Me^pGs and participate in gene activation [11, 12]. These proteins are similar to the MBPs in their DNA binding site (CpG dinucleotide) and differ from them in both their activity (activation versus repression) and in the methylation state of the recognized binding site (C^Un-Me^pG versus C^Me^pG). These findings suggest a third option—methylation may repress gene expression by interfering with the binding of such general activators. The third mechanism is actually a combination of the two known mechanisms; it is similar to the first one in the notion that methylation prevents expression by interfering with the binding of activators and is similar to the second mechanism in the notion that those activators are general (bind to a CpG site). 

In order to examine the relative effects of the two possible global mechanisms (the second and third mechanisms) on the silencing of methylated promoters, we took advantage of two independent recently published genome-wide bisulphite data [13, 14]. Comparison of the expression patterns of genes harboring various amounts of C^Me^pG and of C^Un-Me^pG revealed that the number of C^Un-Me^pGs is strongly associated with gene expression, suggesting an important role for global C^Un-Me^pG binding activators in gene regulation. These results suggest that methylation may repress genes also through interference with the recruitment of general activating agents and not only by the recruitment of repressing agents.

## 2. Results

As stated earlier DNA methylation has two innate results. First it creates C^Me^pGs (which can serve as templates for methyl binding proteins), and second, it eliminates C^Un-Me^pGs (which are the binding sites of various CXXC proteins). To test which of these two optional methylation outcomes has a stronger effect on gene silencing, we developed a bioinformatics approach that allowed us to distinguish between those two outcomes of the methylation process. To this end, we used recent methylome data that measured the degree of methylation of every CpG in the human genome in three cell lines—embryonic stem cells (ES), primary fibroblasts (F), and fibroblasts derived from ES cells (ES-F) [14]. For each tissue, we compared the expression levels of various groups of genes sharing certain promoter features. The analyses were performed separately for genes with and without CpG islands (CGI), using two alternative definitions of CGI (see Methods), in their promoter sequence. 

**Figure 1:**
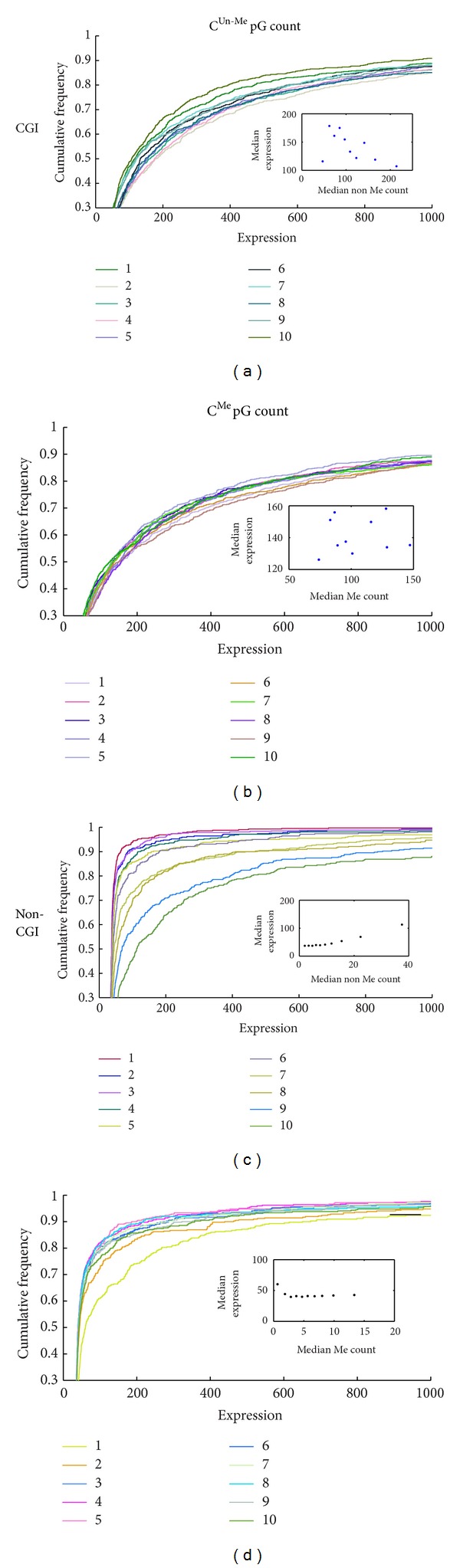
Association between expression levels and promoter methylation. All genes in ES cells were divided into CpG island (a, b) and nonisland (non-CGI) (c, d) genes. The genes in each category were sorted according to the number of C^Un-Me^pGs (a, c) and C^Me^pGs (b, d) in their promoter sequence, and the sorted lists were divided into 10 equally sized bins (765 and 429 genes in each bin of the CGI and non-CGI, resp.). Genes in each bin were resorted according to their expression level and the cumulative distribution is shown. The bins are numbered according to the increasing number of C^Un-Me^pGs (a, c) and C^Me^pGs (b, d). The insert in each graph presents the median expression value plotted as a function of the median methylation level in each bin for C^Un-Me^pGs and C^Me^pGs. Spearman Rho and *P* values were not significant in (a), (b), and (d) (*r* = −0.5,  *P* = 0.133, *r* = 0.067, *P* = 0.865, and *r* = −0.04, *P* = 0.919 resp.) and were highly significant (*r* = 0.952,  *P* = 0.000012) in (c). Note that the gradual increase in expression in association with a gradual increase in C^Un-Me^pG counts is seen in the medians, and thus it is not likely that it is the result of a contamination of nonannotated CGI genes (see Discussion). Essentially the same results were obtained with a different set of methylome and transcriptome data [13] (Figure S12).

Using the methylome data of human ES cells we counted the number of C^Me^pGs and of C^Un-Me^pGs in all promoters (1000 bp upstream to the TSS; see Methods for details). Genes were divided into two categories—CGI genes (those containing a CGI in their promoter) and non-CGI genes. These two gene groups were further sorted according to the number of C^Me^pGs or C^Un-Me^pGs and divided into 10 bins each with an equal amount of genes and a relatively uniform number of C^Me^pGs or C^Un-Me^pGs in the promoter region. For each such bin we plotted the cumulative distribution of the expression levels as measured by Laurent et al. [14] (Figure 1).

Comparison of the expression distribution in the ten bins revealed a clear difference between CGI and non-CGI genes. While in CGI genes there were no significant correlations between neither C^Me^pG counts nor C^Un-Me^pGs counts and expression levels (Figures 1(a) and 1(b); *P* = 0.133 and *P* = 0.865, resp.), in non-CGI genes the number of C^Un-Me^pGs was highly correlated with gene expression (Figure 1(c); *P* = 0.000012). Surprisingly, this correlation was restricted to the C^Un-Me^pG counts and no correlation was observed between C^Me^pGs and gene expression (Figure 1(d); *P* = 0.919). These results were insensitive to the actual definition of CGI, since we got essentially the same results while excluding all non-CGI promoters that were annotated as containing unmethylated regions (UMR) by Straussman et al. [15]. Moreover, the existence of a small set of additional nonannotated CGI cannot influence the results since we have used nonparametric statistics which is more robust toward the presence of outliers. 

**Figure 2:**
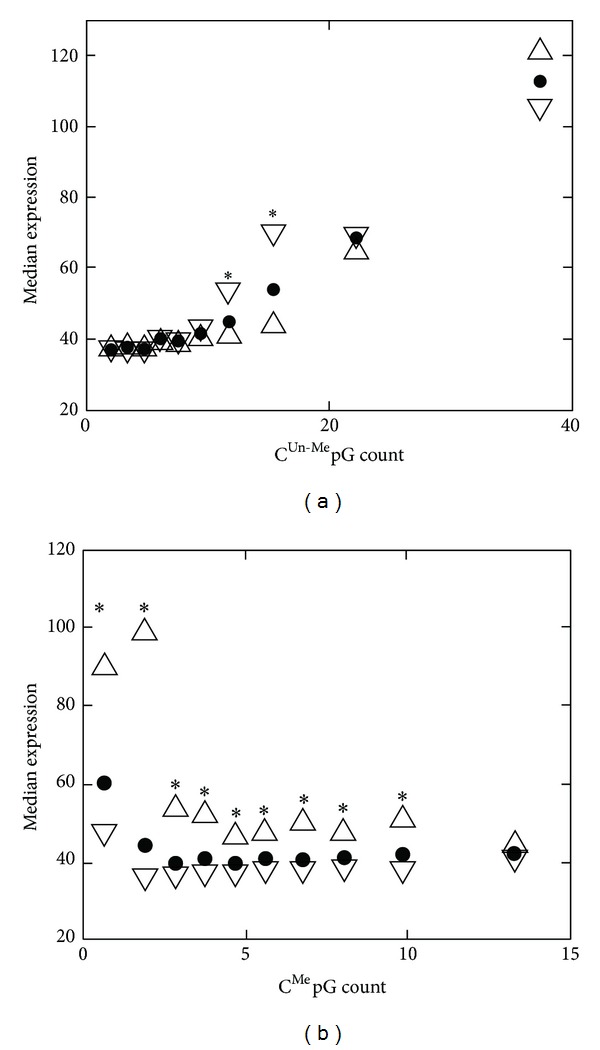
Association between expression levels and promoter methylation status in non-CGI genes. (a) The median expression values of the ten bins shown in Figure 1(c) are plotted as a function of the median C^Un-Me^pG count in each bin (black circle). Each bin is further divided into two halves according to C^Me^pG count, and the median expressions of the genes with high and low methylation levels are represented by up and down pointing triangles, respectively. (b) same as (a), but the bins are divided according to the number of C^Me^pGs (as in Figure 1(d)), and the up and down pointing triangles represent the median expression levels of the genes with high and low C^Un-Me^pG counts. Bins in which the difference in the expression pattern between the two subbins was significant (*P* < 0.001; Mann-Whitney one side test) are marked by an asterisk. The error bars were omitted for clarity and the full distribution of the expression levels of the two groups in each bin in (a) and (b) is presented in Figures S1 and S2, respectively. Essentially the same results were obtained with a different set of methylome and transcriptome data [13] (Figure S13).

 In order to further characterize C^Un-Me^pG contribution to gene expression in non-CGI genes, we decided to isolate its effect from the effect of C^Me^pG. To this end we divided each of the ten bins described earlier (Figure 1(d)) into two equally sized groups according to the number of the C^Un-Me^pGs and compared the expression profiles of the genes in these two subbins. We found for all methylation levels (all ten bins) that a higher number of C^Un-Me^pGs is associated with higher expression level (Figure 2(b) and Figure S2 available online at http://dx.doi.org/10.1155/2013/785731). The reciprocal experiment, in which each bin of the C^Un-Me^pGs (Figure 1(c)) was divided into two according to the number of the C^Me^pGs, revealed that the contribution of the methylation *per se* is restricted to only a few bins (Figures 2(a) and S1).

Similar conclusions were derived from analyzing the effect of the number of CpGs on gene expression when the methylation percentage is kept constant. Binning the data according to the methylation percentage keeps, in each bin, the relative amount of methylated and unmethylated CpGs equal. Each bin with a fixed percentage of methylation was further divided into two subgroups according to the CpG count. Thus in each bin, the subgroup with high CpG count contains both more C^Me^pGs and C^Un-Me^pGs whereas the lower CpG count subgroup contains less C^Me^pGs and less C^Un-Me^pGs. Here again we found statistically significant higher expression levels in higher CpG count subgroup for most of the bins (Figure S3), indicating that the number of unmethylated CpGs and not the number of the methylated CpGs is associated with gene expression. 

The same analyses were performed on the methylome and expression data of the primary fibroblasts and the ES-F cells [14] and on independent methylome and transcriptome datasets [13] and the results were essentially the same (Figures S4–S13). 

These results were not sensitive to the binning method and essentially the same results were obtained using a different binning scheme in which the methylation span (1.8 and 2 for C^Me^pGs and C^Un-Me^pGs, resp.) in each bin was constant (see Methods and Figures S14–S16). 

## 3. Discussion

During the last twenty years it became clear that methyl binding proteins (MBPs) can repress gene expression by turning the chromatin into a repressed structure [16, 17]. However, there is not enough compelling evidence that these proteins serve as part of a major mechanism that ensures the silencing of methylated genes *in vivo*. Recently, a new group of proteins was identified [11, 12]. These proteins are specifically recruited to unmethylated DNA (through their CXXC domain) and participate in opening the chromatin around it. This finding raises the interesting possibility that DNA methylation may repress gene expression through blocking the binding of these or similar proteins.

By comparing the expression level of genes that were grouped according to the number of C^Me^pGs or C^Un-Me^pGs in their promoter sequence (Figures 1 and 2) we were able to conclude that the number of C^Un-Me^pGs is highly associated with gene expression, much more than the association of the number of C^Me^pGs with gene repression in non-CGI genes. These results suggest that C^Un-Me^pG binding activators are involved in the activation of nonmethylated genes and that their contribution may be bigger than the contribution of MBPs to gene repression. This observation challenges the widely accepted view that DNA methylation represses gene expression by recruiting methyl specific repressors. Interestingly, such association was not found in CGI genes probably because in CGI the number of C^Un-Me^pGs exceeds a certain threshold and the activity of the C^Un-Me^pG binding proteins is already saturated. 

It should be noted that our analysis does not address the specific mechanism of such activators recruitment. It might be that a single C^Un-Me^pG is sufficient for their recruitment and therefore promoters with higher C^Un-Me^pG count are more active. But it can also be that a small region with high local density of C^Un-Me^pG (such as found in ICP regions—[18]) is required for the recruitment of activators, and thus regions with higher C^Un-Me^pG counts have higher chances for the occurrence of such C^Un-Me^pG clusters.

Recently, Stadler et al. [19] have shown that low-methylated regions (LMRs) are characterized by the presence of DNA-binding factors and their binding is necessary and sufficient to create LMRs [19]. This finding suggests that expression may be the cause rather than the consequence of low methylation. Is this hypothesis sufficient to explain our findings as well? This is unlikely to be the case since we have analyzed the data in bins with a fixed number of C^Me^pGs (Figure 2(b)). In a certain bin all genes have the same methylation level, and thus the association between expression and the number of C^Un-Me^pGs is actually a correlation with the number of total CpG count in the promoter (since #CpG = #C^Me^pG + #C^Un-Me^pG and the #C^Me^pG is fixed in the bin). Thus expression can influence the C^Un-Me^pGs only by changing the genomic content (#CpG) which is a very unlikely scenario. 

Although we have excluded annotated CGI genes from our analysis, the results may be influenced from the contamination of nonannotated CGI in certain bins. Indeed, the precise definition of an island is far from being simple and the sequence based definition we have used [20, 21] failed to annotate many genomic regions that are protected from methylation [2, 15, 21]. Nevertheless, three lines of evidence suggest that our results demonstrate a real effect of  C^Un-Me^pGs on non-CGI genes rather than the consequence of nonannotated CGI. First, a different definition of islands which is based on short sequences that are characteristic of unmethylated regions (UMRs) identified 3694 constitutively unmethylated genomic regions [15]. Nevertheless, most of these UMRs are not in promoter regions, and thus the use of this alternative definition added only 260 genes (<3.5%) to the CGI group. Second, we have repeated our analyses after excluding those genes and got essentially the same results. Finally, the association between C^Un-Me^pG counts and expression levels was found to be significant using nonparametric statistical tests (Spearman correlation of the median in Figure 1 and Mann-Whitney test in Figure 2). Such nonparametric tests are not sensitive to outliers and can be biased by unannotated islands only if more than half of the promoters in each bin were unannotated. This is not a reasonable scenario regardless of the specific CGI definition chosen. 

Although most of the direct evidence connecting MBP to gene silencing by DNA methylation comes from *in vitro* experiments, a few experiments demonstrated its importance *in vivo *[16]. For example, it was demonstrated that the repression of a methylated construct (introduced to cells by transfection) is reduced in F9 cells lacking Mecp-1 [22]. Our results do not contradict this finding since our data do not exclude the effect of MBPs as repressors but reveal additional effect of potential C^Un-Me^pG binding activators. Indeed the methylated constructs that were introduced to F9 cells lacking MeCP-1 showed partial repression, supporting our claim that other mechanisms, in addition to repression by MBPs, are involved in methylation mediated gene repression. 

We have explained the higher levels of gene expression for genes with high C^Un-Me^pG count (Figure 1) by the involvement of C^Un-Me^pG binding proteins in gene activation. An alternative explanation may be the sensitivity of the MBPs to the density of the methyl groups. Indeed it was shown that repression by MeCP2 depends on the density of methylation [23], and a recent paper showed that it is due to the cooperative nature of MeCP2 binding to the DNA [24]. However, this latter explanation falls short in explaining the results presented in Figures 2(a) and S1 since the increase in the number of C^Me^pGs should result in increase of methyl density but, nevertheless, did not reduce expression in many cases. Moreover, our finding that in promoters with a constant percentage of methylation the number of unmethylated CpGs shows higher effect on expression than the number of methylated CpGs (Figure S3) cannot be explained merely by cooperativity. 

Which are the C^Un-Me^pG binding proteins responsible for gene activation? Recent studies have identified two proteins, KDM2A and CFP1, that bind C^Un-Me^pG mainly in CGI and are involved in gene activation [11, 12]. Our analyses suggest a role for C^Un-Me^pG binding activators in the regulation of non-CGI genes. Obvious candidates are the KDM2A and CFP1 proteins since genome-wide binding data reveals that they bind also to some extent outside of CGIs (66% of KDM2A and 7.4% of Cfp1 clusters do not overlap with a CGI [11, 12]). However, other, not yet identified, C^Un-Me^pG binding proteins may also contribute to the activation of the not methylated non-CGI genes. Further analysis of KDM2A knockdown expression data revealed that KDM2A alone cannot explain this activation. We have repeated our analyses using expression data (GSE21201) from cells with normal and reduced levels of KDM2A [11] and found that in both cases high levels of C^Un-Me^pG are associated with high expression levels (Figures S17-S18). 

**Figure 3:**
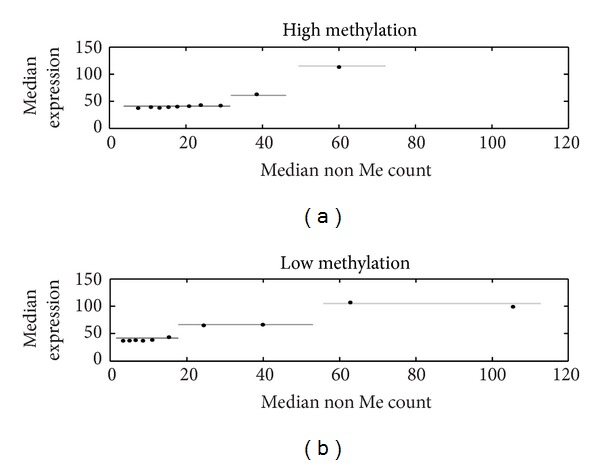
Interplay between C^Me^pG and C^Un-Me^pG levels. (a) The median expressions of ten bins of C^Un-Me^pG were plotted separately for promoters with high (above median) and low (below median) methylation levels. The expression levels of the genes fell into three categories (high, middle, and low) schematically labeled by lines with different grayscale. Dividing the data into 20 bins gives the same results (Figure S19).

What is the interplay between MBP mediated repression and C^Un-Me^pG binding proteins mediated activation? We found strong association of C^Un-Me^pG counts and active genes in almost all levels of methylation (Figure 2(b)). We also found that C^Me^pG counts were associated with gene repression only in genes harboring moderate levels of C^Un-Me^pG (between 8 and 18; Figure 2(a)). The expression level is not determined simply by the ratio of C^Un-Me^pG and C^Me^pG as demonstrated by the analysis of the methylation percentage (Figure S3). We propose that in promoters with high levels of C^Un-Me^pG the activity of the C^Un-Me^pG binding activators is saturated and this is probably the reason for the lack of correlation between C^Un-Me^pG counts and expression in CGI genes (Figure 1). On the other hand, in promoters with lower levels of C^Un-Me^pG the actual expression level is achieved through a balance between the activity of methyl binding repressors and C^Un-Me^pG binding activators. Although C^Un-Me^pGs seem to affect expression in almost all levels of C^Me^pG, their actual contribution is different in promoters with high and low methylation levels (Figure 3).

## 4. Conclusions

**Figure 4:**
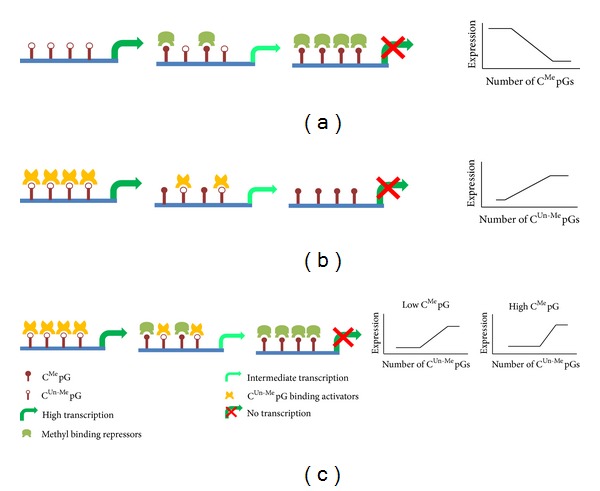
Three potential models, describing the association between methylation and gene regulation. (a) Methyl binding repressors based model. (b) C^Un-Me^pG binding activators based model. (c) A combined model. The schematic graphs on the right represent the expected results according to each model.

We found a correlation between the number of C^Un-Me^pGs and gene expression suggesting that the association of methylation and gene expression cannot be explained solely by the activity of methyl binding repressors (Figure 4(a)). On the other hand, C^Un-Me^pG binding proteins alone (Figure 4(b)) cannot explain the different expression levels observed for promoters that differ only in their methylation levels. Taken together, our results suggest a combined model (Figure 4(c)) in which both C^Me^pG binding repressors and C^Un-Me^pG binding activators have a role in achieving the desired transcription levels. It should be noted that the balance between these two processes is important only in non-CGI genes, probably because in CGI the number of C^Un-Me^pGs exceeds a certain threshold. Further experiments are required to identify the proteins that are involved in the activation of C^Un-Me^pG-containing genes and for deciphering the interplay between those proteins and the MBPs in the regulation of gene expression. 

## 5. Methods

### 5.1. Study Design

The study follows the following design: (1) data collection; (2) promoter identification; (3) CGI identification; (4) counting the number of C^Me^pG and C^Un-Me^pG in each promoter; (5) Binning the promoters according to the number of C^Me^pG and C^Un-Me^pG; (6) association of each bin with the expression data; (7) statistical assessment of the results. 

### 5.2. Datasets

Methylation data for three cell types—WA09 hESC (ES), hESC-derived fibroblasts (ES-F), and neonatal fibroblast line (F)—were downloaded from GEO (GSE19418) [14]. These data include, for each genomic location, the number of methylated and unmethylated reads. We used this information to calculate for each CpG in the human genome (based on the NCBI36/hg18 release) its methylation level (number of methylated reads divided by the total reads). 

For each promoter region (defined as the 1000 bps upstream to the transcription start site (TSS) based on UCSC genome browser annotations) the C^Me^pG count was defined as the sum of the methylation level of all CpGs in the promoter (two strands average was used). The methylation level of nonsequenced residues was calculated by extrapolation of the average methylation levels of sequenced CpGs of the same promoter. The number of unmethylated CpGs was calculated similarly. We included in our analysis only genes that have sequence reads for sufficient percentage (>50%) and amounts (≥4) of CpGs. Expression data were also taken from [14], for the three cell types. The methylome data and the expression data were integrated based on genomic location. Genes with ambiguous location data were excluded from further analyses. Overall we had both methylation and expression data for 13142, 13770, and 14389 genes for ES, ES-F, and fibroblasts, respectively.

We have also used the methylome and transcriptome data of human ES h1 cells from [13]. The data were analyzed exactly in the same manner resulting in 9478 genes with methylation and expression data. FPKM values (as calculated by Cufflink [25]) for each gene were used as the expression metric. 

Expression data of KD2MA knockdown was taken from Blackledge et al. [11]. 

### 5.3. CpG Island (CGI) Definition

We have further divided all genes into two groups—CGI and non-CGI genes. We used the common CGI definition used in the UCSC genome browser [20] and defined as CGI gene every gene in which greater than 10% of its promoter region overlaps with a CGI. Non-CGI genes were defined as genes with no overlap between the promoter region and a CGI. Using this definition, 7656, 7896, and 8208 genes were defined as CGI genes and 4290, 4635, and 4889 as non-CGI genes in ES, ES-F, and fibroblasts, respectively. In order to be sure that the non-CGI group does not contain many nonannotated CGI we have repeated the analyses after omitting from the non-CGI group all genes with a constitutively unmethylated region (UMR) in their promoter region according to [15]. 

### 5.4. Binning Methods

All genes were ranked three times, each time according to one of the following features: (i) the total number of methyls (C^Me^pGs) in their promoter sequence; (ii) the total number of unmethylated CpGs (C^Un-Me^pGs) in their promoter sequence; (iii) the percentage of methylation in their promoter sequence. Each ranked list was divided into ten bins with the same number of genes in each bin. For the ES data we also implemented a different binning method in which the methylation span was kept constant (1.8 and 2 for C^Me^pGs and C^Un-Me^pGs, resp.) instead of the number of genes. Each of these bins was further divided into two equally sized subgroups according to the median of C^Un-Me^pGs, C^Me^pGs, and CpG count, respectively. We plotted the cumulative distribution of the expression levels of all the bins.

### 5.5. Statistical Analysis

The statistical significance of the difference in expression level among the ten bins that differ in their methylation levels (Figure 1) was tested using Spearman correlation on the median expression values in each bin. We used the median value as a representative for each group in order to avoid the influence of outliers. 

Assessment of the significant difference between the two sub groups in each bin (Figure 2) was done with the Mann-Whitney test using Matlab ranksum function. 

## Supplementary Material

In order to strengthen the argument demonstrated in figure 2 we present the cumulative distributions of all bins separated according to CMepG count, CUn-MepG and CMepG percentage in figures S1, S2 and S3 respectively. The same results were obtained also in primary fibroblast cells (Figures S4-S7) and in ES-F cells (Figures S8-S11). The data was also insensitive to the source of the methylome data since we got essentially the same results using a different methylome data (Figures S12-S13). The results were also insensitive to the binning method since using a different binning method gave essentially the same results (Figures S15-S16)
Repeating the experiment using expression data from cells containing either WT levels or KD KDM2a (Blackledge et al., 2010) reveals that KDM2a is not responsible for the higher expression in promoters with many CUn-MepG (Figures S17-S18).
Figure S19 is similar to Figure 4 using 20 bins instead of 10 bins.
