# Supplementary material for: Integrative Analysis of Methylome and Transcriptome Reveals the Importance of Unmethylated CpGs in Non-CpG Island Gene Activation
Source: Biomed Res Int. 2013 Jul 10;2013:785731. doi: 10.1155/2013/785731 (PMC3722964; doi:10.1155/2013/785731)

## Supplementary data

### The effect of different binning methodology

We have used two binning methods. In both methods we started by ranking the genes according to their methylation levels and divided them into ten groups. In the first method (used in Figures 1-4 and S1-S9) the number of genes in each bin was kept constant, whereas in the second method (used in Figures S10-S12) the methylation span was constant (1.8 and 2 for $C^{Me}$pGs and $C^{Un\text{-}Me}$pGs respectively). The results were essentially the same with minor changes between the binning methods in the actual range in which the effect of the $C^{Me}$pGs and $C^{Un\text{-}Me}$pGs were seen. For example, the effect of $C^{Me}$pGs was seen for $C^{Un\text{-}Me}$pGs levels of 8-18 using the first binning method and 6.5-18.5 using the second method.

### Supplementary figure legends

**Figure S1 -** ***Association between $C^{Me}$pG count and gene expression levels***

*Shown are the cumulative distributions of the expression levels of genes in ES cells in ten bins (each containing 420 genes) of the $C^{Un\text{-}Me}$pG, each divided into two equal subsets according to the number of the $C^{Me}$pGs. The subsets with lower $C^{Me}$pGs counts are plotted with solid lines and those with higher values with dotted lines. The range of $C^{Me}$pGs in each bin and the P value (Mann-Whitney; one side) comparing the expression levels of the two subsets are shown for each graph.*

**Figure S2 -** ***Association between $C^{Un\text{-}Me}$pG count and gene expression levels***

*Shown are the cumulative distributions of the expression levels of genes in ES cells in ten bins (each containing 420 genes) of the $C^{Me}$pG, each divided into two equal subsets according to the number of the $C^{Un\text{-}Me}$pGs. The subsets with lower $C^{Un\text{-}Me}$pGs counts are plotted with solid lines and those with higher values with dotted lines. The range of $C^{Me}$pGs in each bin and the P value (Mann-Whitney; one side) comparing the expression levels of the two subsets are shown for each graph.*

**Figure S3 - *Association between $C^{Me}$pG percentage and gene expression level in ES cells***

*Shown are the cumulative distributions of the expression levels of genes in ES cells in ten bins (each containing 420 genes) of the $C^{Me}$pG percentage, each divided into two equal subsets according to the number of the CpGs. The subsets with lower CpG count are plotted with solid lines and those with higher values with dotted lines. The range of CpG percentage in each bin and the P value (Mann-Whitney; two sided) estimating the statistical significance of the difference between the two curves are shown for each graph.*

***Figure S4– Association between expression levels and promoter methylation in non CGI genes in primary fibroblast cells***

*All CpG island genes in primary fibroblasts  cells were divided into 10 equally sized bins according to the number of $C^{Un\text{-}Me}$ pGs(A) and $C^{Me}$ pGs (B) ) in their promoter sequence. Cumulative distributions of the expression data are shown. The bins are numbered according to the increasing number of $C^{Un\text{-}Me}$ pGs (A) and $C^{Me}$ pGs (B). The median*

*expression value is plotted as a function of the median methylation level in each bin for $C^{Un\text{-}Me}$ pGs(C) and $C^{Me}$ pGs (D). Spearman Rho and P values are presented.*

***Figure S5 – Association between $C^{Un\text{-}Me}$pG count and gene expression levels in primary fibroblast cells***

*Shown are the cumulative distributions of the expression levels of genes in primary fibroblast cells in ten bins (each containing 480 genes) of the $C^{Me}$pG, each divided into two equal subsets according to the number of the $C^{Un\text{-}Me}$pGs. The subsets with lower $C^{Un\text{-}Me}$pGs counts are plotted with solid lines and those with higher values with dotted lines. The range of $C^{Me}$pGs in each bin and the P value (Mann-Whitney; one sided) estimating the statistical significance of the difference between the two curves are shown for each graph.*

**Figure S6 -** ***Association between $C^{Me}$pG count and gene expression level in primary fibroblast cells***

*Shown are the cumulative distributions of the expression levels of genes in ES cells in ten bins (each containing 480 genes) of the $C^{Un\text{-}Me}$pG, each divided into two equal subsets according to the number of the $C^{Me}$pGs. The subsets with lower $C^{Me}$pGs counts are plotted with solid lines and those with higher values with dotted lines. The range of $C^{Un\text{-}Me}$pGs in each bin and the P value (Mann-Whitney; one sided) estimating the statistical significance of the difference between the two curves are shown for each graph.*

**Figure S7 -** ***Association between $C^{Me}$pG percentage and gene expression level in primary fibroblast cells***

*Shown are the cumulative distributions of the expression levels of genes in primary fibroblast cells in ten bins (each containing 480 genes) of the $C^{Me}$pG percentage, each divided into two equal subsets according to the number of the CpGs. The subsets with lower CpG count are plotted with solid lines and those with higher values with dotted lines. The range of CpG percentage in each bin and the P value (Mann-Whitney; two sided) estimating the statistical significance of the difference between the two curves are shown for each graph.*

**Figure S8 – Association between expression levels and promoter methylation in non CGI genes in ES-F cells**

*All CpG island genes in ES-F cells were divided into 10 equally sized bins according to the number of $C^{Un\text{-}Me}$ pGs(A) and $C^{Me}$ pGs (B) ) in their promoter sequence. Cumulative distributions of the expression data are shown. The bins are numbered according to the increasing number of $C^{Un\text{-}Me}$ pGs (A) and $C^{Me}$ pGs (B). The median expression value is plotted as a function of the median methylation level in each bin for $C^{Un\text{-}Me}$ pGs(C) and $C^{Me}$ pGs (D). Spearman Rho and P values are presented.*

**Figure S9 – Association between $C^{Un\text{-}Me}$pG count and gene expression levels in ES-F cells**

*Shown are the cumulative distributions of the expression levels of genes in primary fibroblast cells in ten bins (each containing 460 genes) of the $C^{Me}$pG, each divided into two equal subsets according to the number of the $C^{Un\text{-}Me}$pGs. The subsets with lower $C^{Un\text{-}Me}$pGs counts are plotted with solid lines and those with higher values with dotted lines. The range of $C^{Me}$pGs in each bin and the P value (Mann-Whitney; one sided) estimating*

*the statistical significance of the difference between the two curves are shown for each graph.*

**Figure S10 -** ***Association between $C^{Me}$pG count and gene expression level in ES-F cells***

*Shown are the cumulative distributions of the expression levels of genes in ES cells in ten bins (each containing 460 genes) of the $C^{Un\text{-}Me}$pG, each divided into two equal subsets according to the number of the $C^{Me}$pGs. The subsets with lower $C^{Me}$pGs counts are plotted with solid lines and those with higher values with dotted lines. The range of $C^{Un\text{-}Me}$pGs in each bin and the P value (Mann-Whitney; one sided) estimating the statistical significance of the difference between the two curves are shown for each graph.*

**Figure S11 -** ***Association between $C^{Me}$pG percentage and gene expression level in ES-F cells***

*Shown are the cumulative distributions of the expression levels of genes in ES-F cells in ten bins (each containing 460 genes) of the $C^{Me}$pG percentage, each divided into two equal subsets according to the number of the CpGs. The subsets with lower CpG count are plotted with solid lines and those with higher values with dotted lines. The range of CpG percentage in each bin and the P value (Mann-Whitney; two sided) estimating the statistical significance of the difference between the two curves are shown for each graph.*

***Figure S12 – Association between expression levels and promoter methylation in the Lister et al., data***

*Exactly the same analysis represented in Figure 1 were performed on an independent dataset of human ES (h1) cells published in Lister et al. (2009). Spearman Rho and P values were not significant in A,B and D (r=-0.5,P=0.1; r=0.6, P=0.07 and r=-0.24, P=0.5 respectively) and were highly significant (r=0.95,P= 0.00002 ) in C.*

***Figure S13 – Association between expression levels and promoter methylation status in non CGI genes in the Lister et al., data***

*Exactly the same analysis represented in Figure 2 were performed on an independent dataset of human ES (h1) cells published in Lister et al. (2009). Note that the results are essentially the same.*

***Figure S14 – Association between expression levels and promoter methylation in non CGI genes in ES cells using a different binning method***

*Same as figure 1c and 1d beside the binning method that used a constant methylation span (1.8 and 2 for* $C^{Me}$*pGs and* $C^{Un\text{-}Me}$*pGs respectively) instead of a constant number of genes. The methylation range and the number of genes in each bin are shown in figures S14 and S13 for A and B respectively.*

***Figure S15 – Association between*** $C^{Un\text{-}Me}$***pG count and gene expression levels in ES cells using a different binning method***

*Same as figure S2 beside the binning method that used a constant methylation span (1.8* $C^{Me}$*pGs) instead of a constant number of genes. In each graph we show the number of the genes in the bin (N) the* $C^{Me}$*pGs range and the Mann-Whitney P value.*

**Figure S16 -** ***Association between $C^{Me}pG$ count and gene expression level in ES cells using a different binning method***

*Same as figure S1 beside the binning method that used a constant methylation span (2 $C^{Un\text{-}Me}pGs$) instead of a constant number of genes. In each graph we show the number of the genes in the bin (N) the $C^{Un\text{-}Me}pGs$ range and the Mann-Whitney P value.*

**Figure S17 -** ***Association between $C^{Un\text{-}Me}pG$ count and gene expression levels in the WT data of Blackledge et al., 2010***

*Shown are the cumulative distributions of the expression levels of genes in ES cells(average of the expression data taken from GSM530254 and GSM530255) in ten bins (each containing 710 genes) of the $C^{Me}pG$, each divided into two equal subsets according to the number of the $C^{Un\text{-}Me}pGs$. The subsets with lower $C^{Un\text{-}Me}pGs$ counts are plotted with solid lines and those with higher values with dotted lines. The range of $C^{Me}pGs$ in each bin and the P value (Mann-Whitney; one side) comparing the expression levels of the two subsets are shown for each graph.*

**Figure S18 -** ***Association between $C^{Un\text{-}Me}pG$ count and gene expression levels in the KDM2a KD data of Blackledge et al., 2010***

*Shown are the cumulative distributions of the expression levels of genes in ES cells(average of the expression data taken from GSM530256 and GSM530257) in ten bins (each containing 710 genes) of the $C^{Me}pG$, each divided into two equal subsets according to the number of the $C^{Un\text{-}Me}pGs$. The subsets with lower $C^{Un\text{-}Me}pGs$ counts are plotted with solid lines and those with higher values with dotted lines. The range of*

*$C^{Me}$pGs in each bin and the P value (Mann-Whitney; one side) comparing the expression levels of the two subsets are shown for each graph.*

**Figure S19 - *Interplay between $C^{Me}$pG and $C^{Un\text{-}Me}$pG levels***

*Same as figure 3, beside using 20 bins instead of 10.*

Figure S1

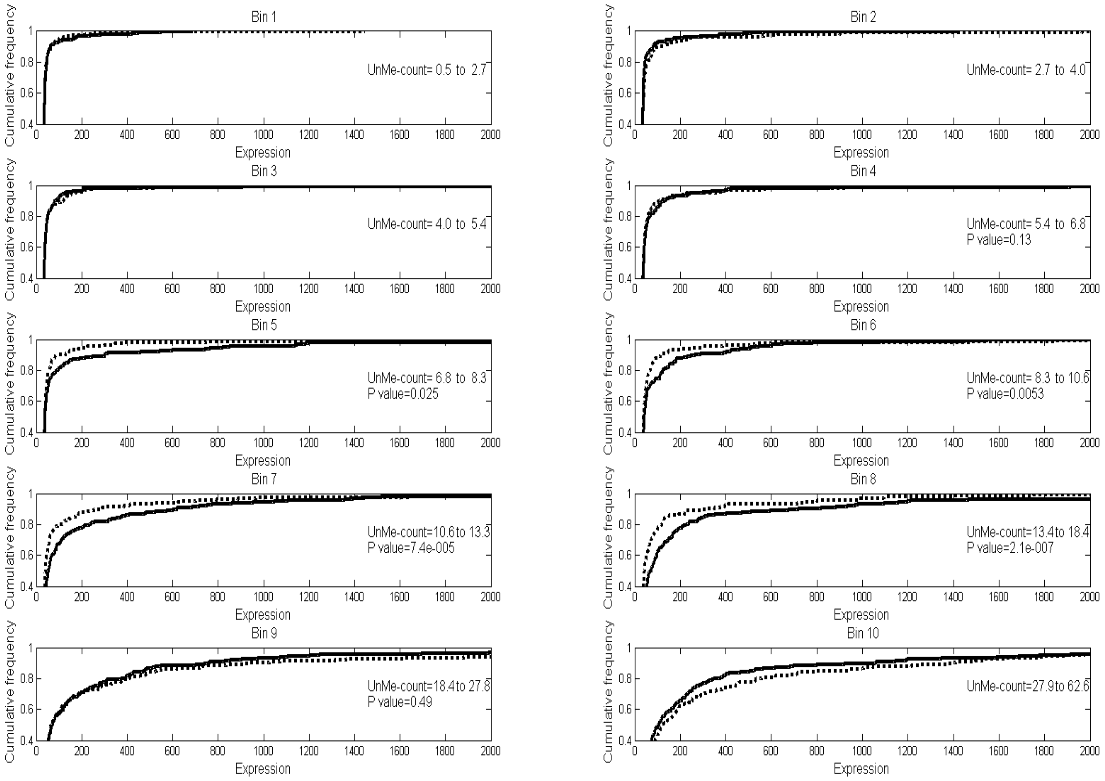

Figure S2

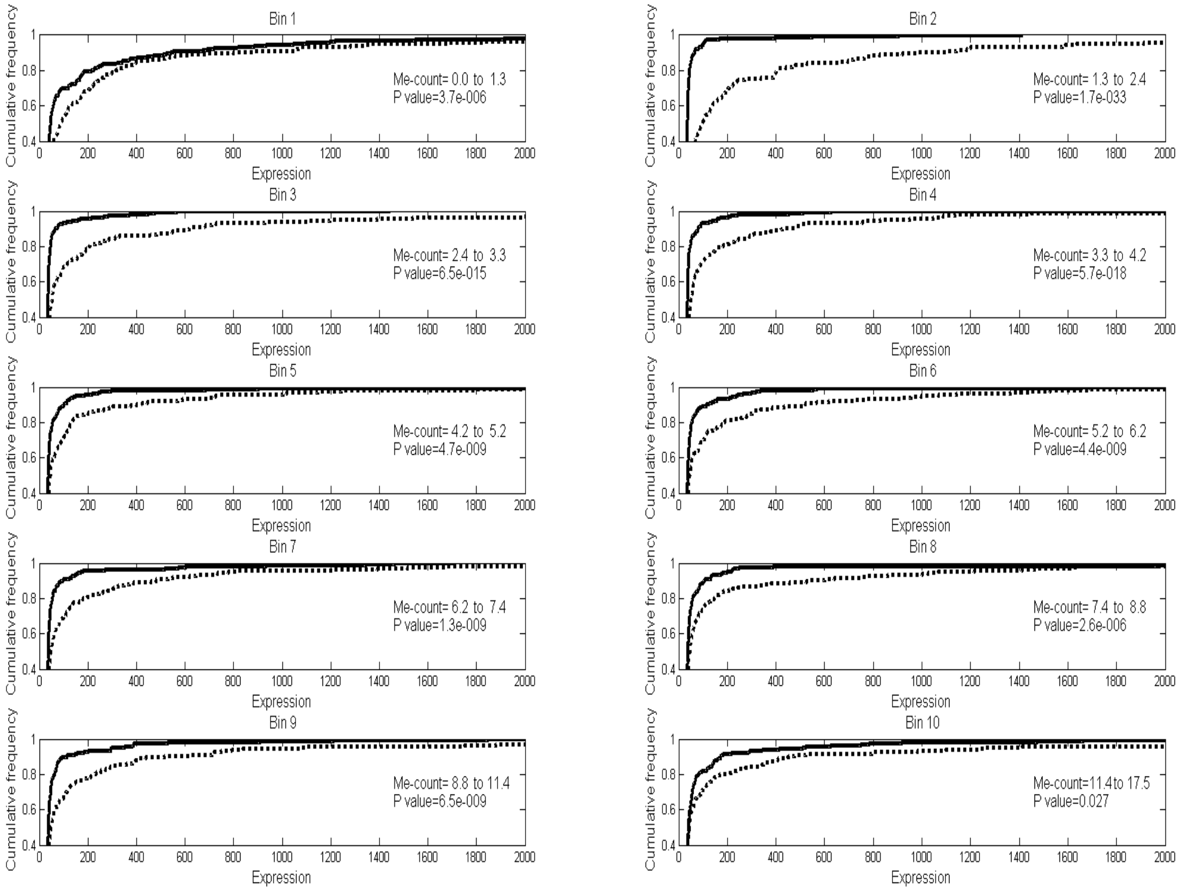
Bin 1
Me-count= 0.0 to 1.3
P value=3.7e-006
Bin 2
Me-count= 1.3 to 2.4
P value=1.7e-033
Bin 3
Me-count= 2.4 to 3.3
P value=6.5e-015
Bin 4
Me-count= 3.3 to 4.2
P value=5.7e-018
Bin 5
Me-count= 4.2 to 5.2
P value=4.7e-009
Bin 6
Me-count= 5.2 to 6.2
P value=4.4e-009
Bin 7
Me-count= 6.2 to 7.4
P value=1.3e-009
Bin 8
Me-count= 7.4 to 8.8
P value=2.6e-006
Bin 9
Me-count= 8.8 to 11.4
P value=6.5e-009
Bin 10
Me-count=11.4 to 17.5
P value=0.027
Cumulative frequency
Expression

Figure S3

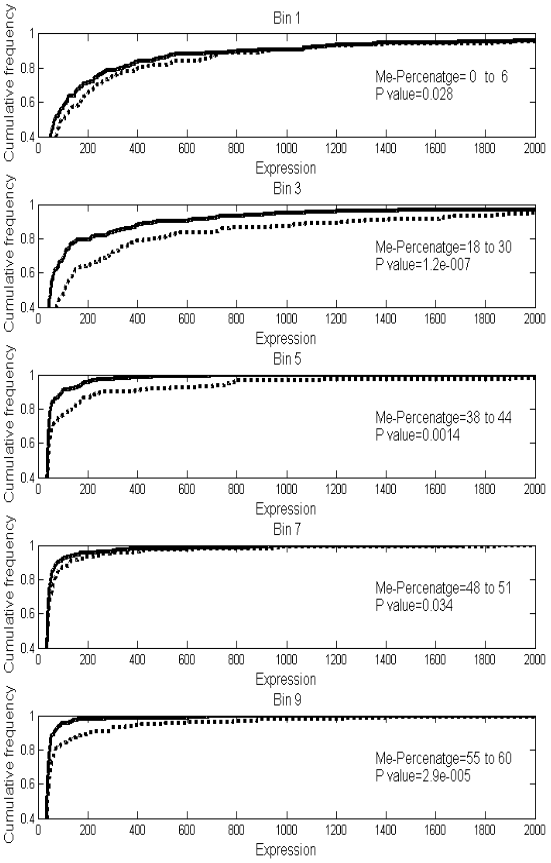

Bin 1
Me-Percenatge= 0 to 6
P value=0.028
Bin 2
Me-Percenatge= 6 to 18
P value=0.00013
Bin 3
Me-Percenatge=18 to 30
P value=1.2e-007
Bin 4
Me-Percenatge=30 to 38
P value=2.6e-015
Bin 5
Me-Percenatge=38 to 44
P value=0.0014
Bin 6
Me-Percenatge=44 to 48
P value=0.0004
Bin 7
Me-Percenatge=48 to 51
P value=0.034
Bin 8
Me-Percenatge=51 to 55
P value=0.14
Bin 9
Me-Percenatge=55 to 60
P value=2.9e-005
Bin 10
Me-Percenatge=60 to 70
P value=0.034
Cumulative frequency
Expression

Figure S4

Fibroblasts

$C^{Un\text{-}Me}pG$ count

$C^{Me}pG$ count

A

B

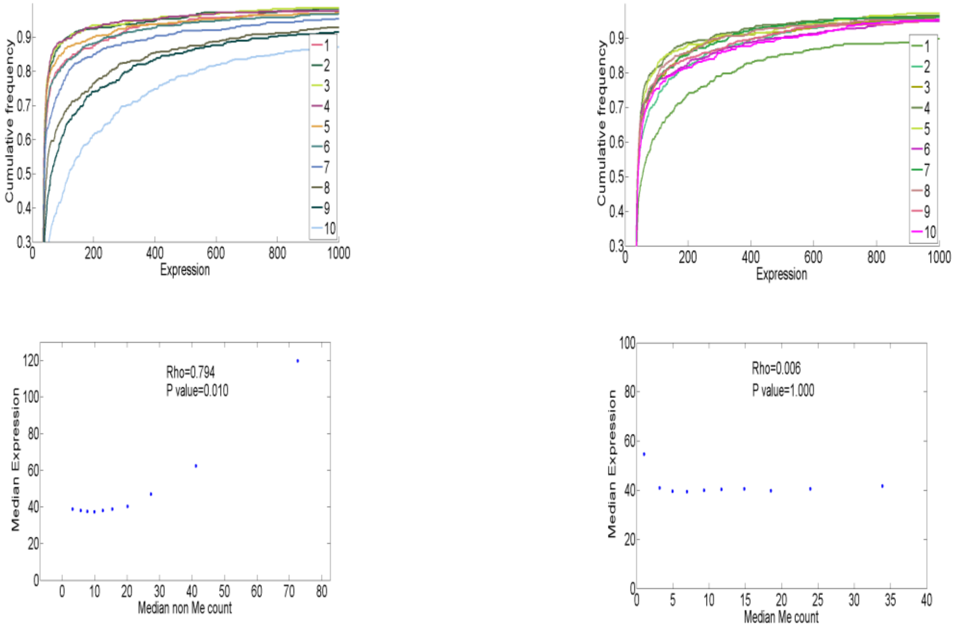

Figure S5

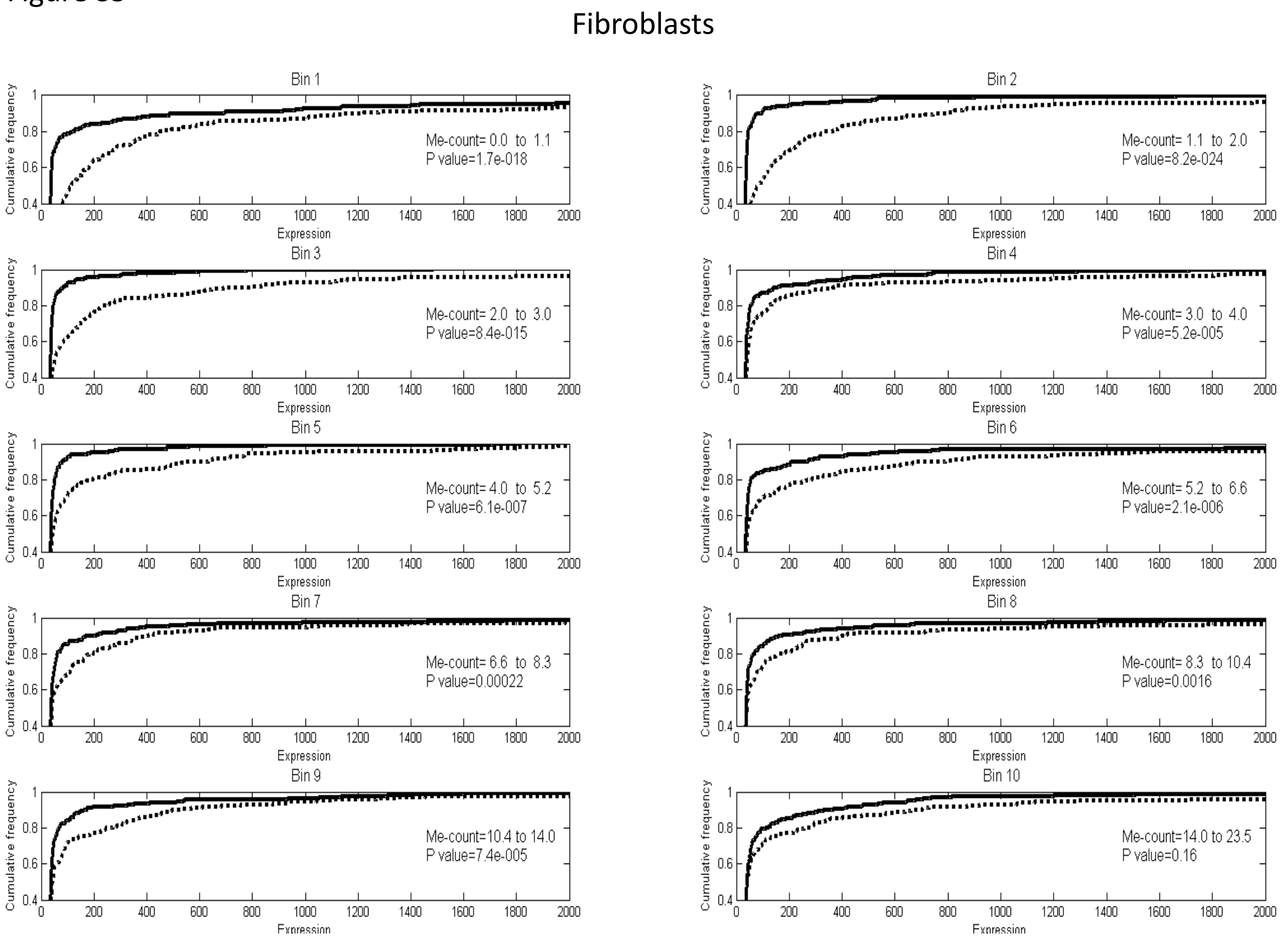
Fibroblasts
Bin 1
Me-count= 0.0 to 1.1
P value=1.7e-018
Bin 2
Me-count= 1.1 to 2.0
P value=8.2e-024
Bin 3
Me-count= 2.0 to 3.0
P value=8.4e-015
Bin 4
Me-count= 3.0 to 4.0
P value=5.2e-005
Bin 5
Me-count= 4.0 to 5.2
P value=6.1e-007
Bin 6
Me-count= 5.2 to 6.6
P value=2.1e-006
Bin 7
Me-count= 6.6 to 8.3
P value=0.00022
Bin 8
Me-count= 8.3 to 10.4
P value=0.0016
Bin 9
Me-count=10.4 to 14.0
P value=7.4e-005
Bin 10
Me-count=14.0 to 23.5
P value=0.16
Cumulative frequency
Expression

Figure S6

Fibroblasts

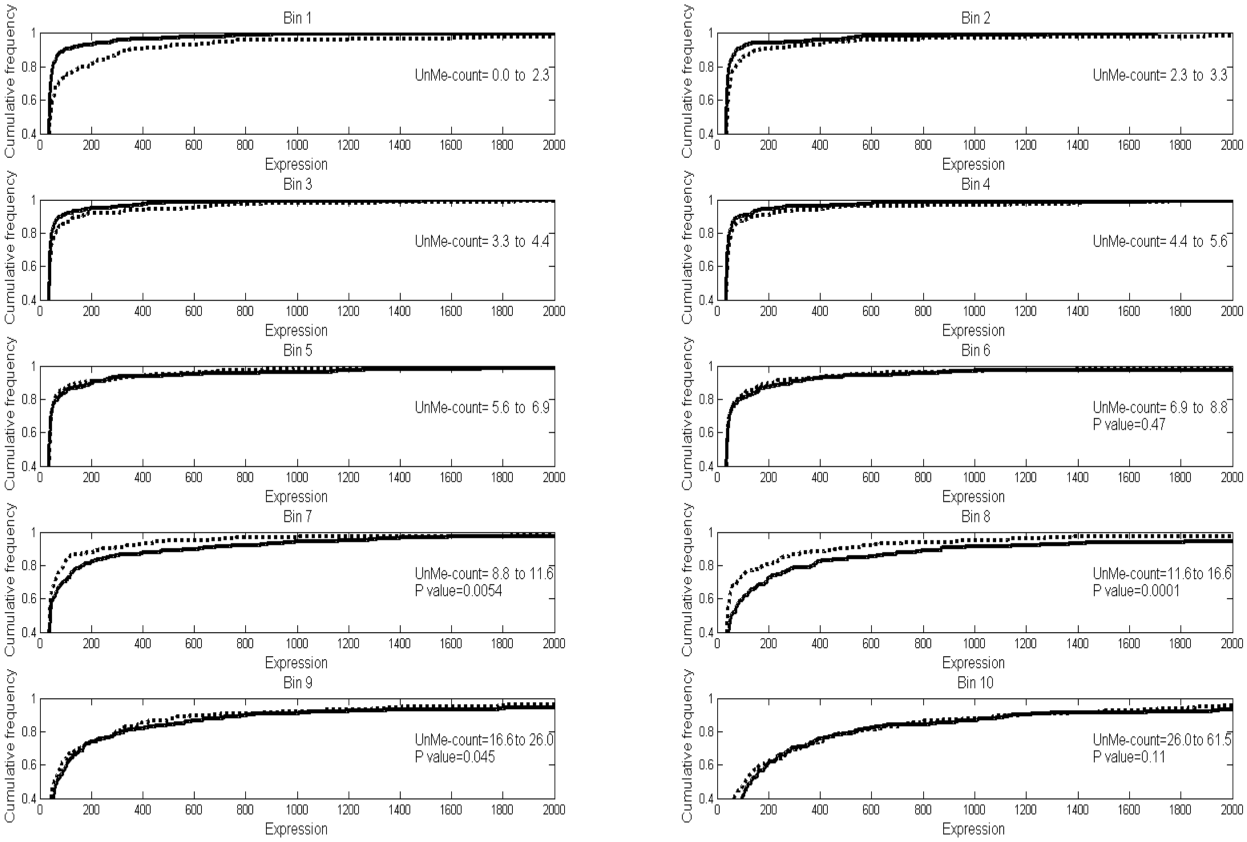

Figure S7

Fibroblasts

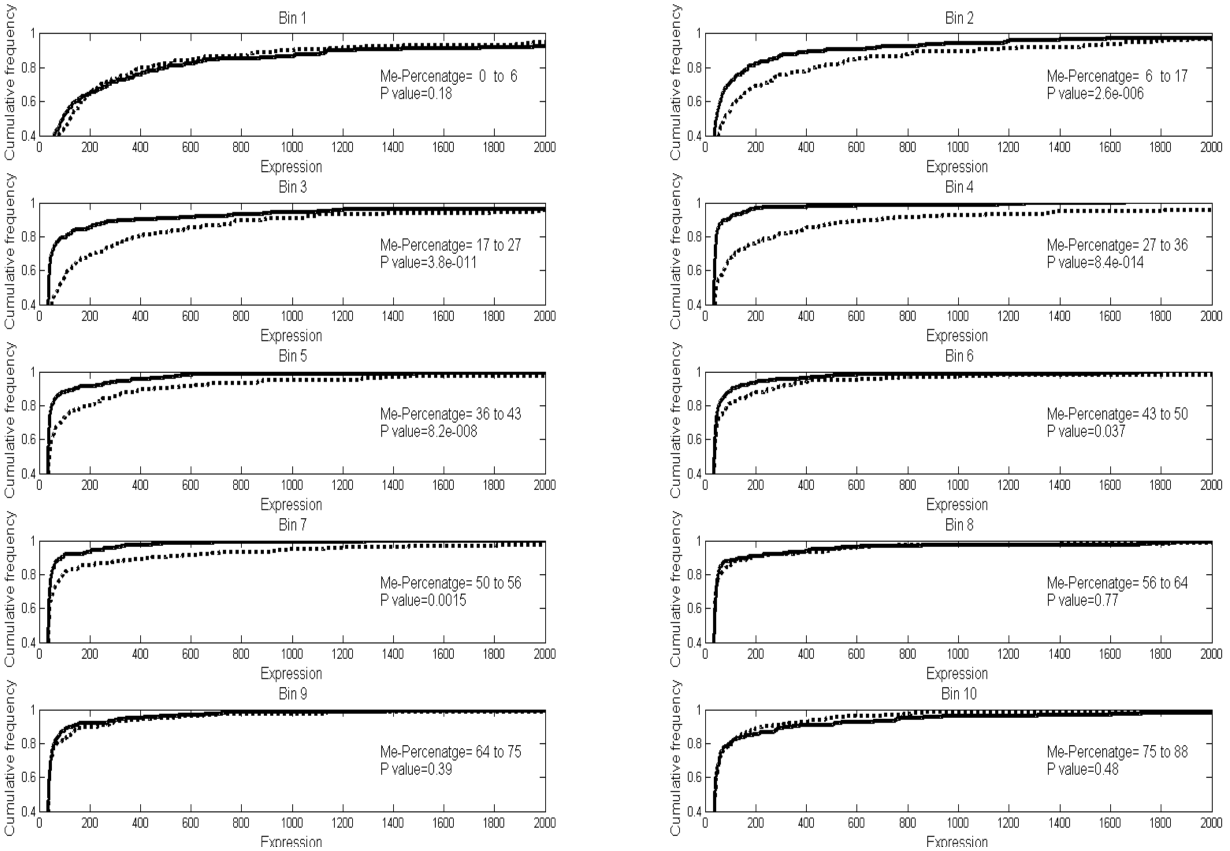

Figure S8

ES_F

$C^{Un-Me}pG$ count $C^{Me}pG$ count

A

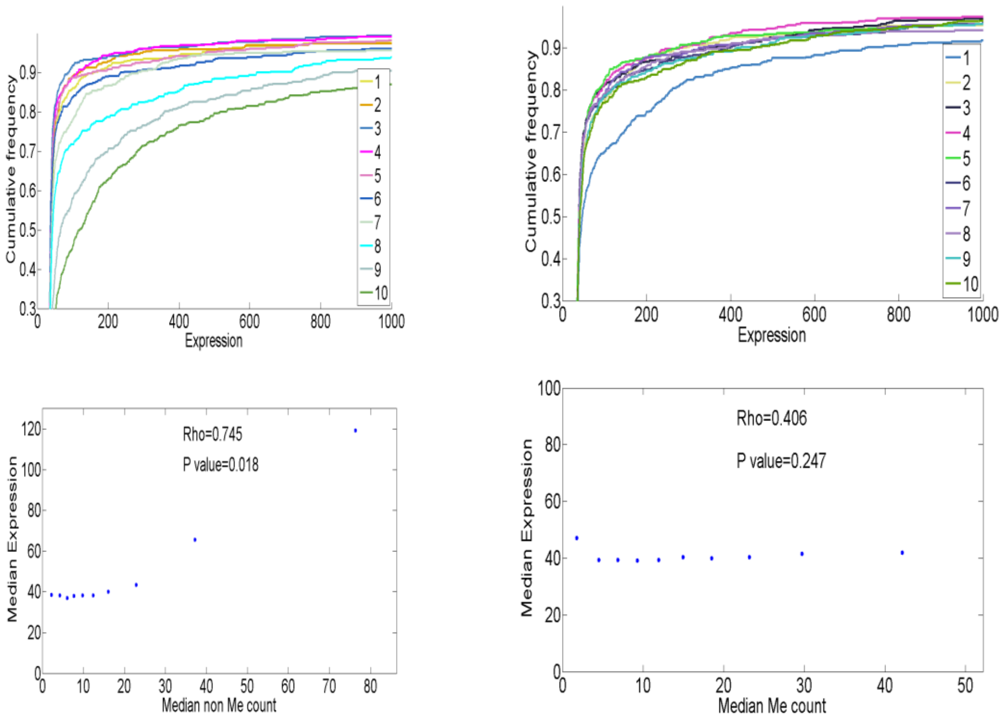

Figure S9

ES_F

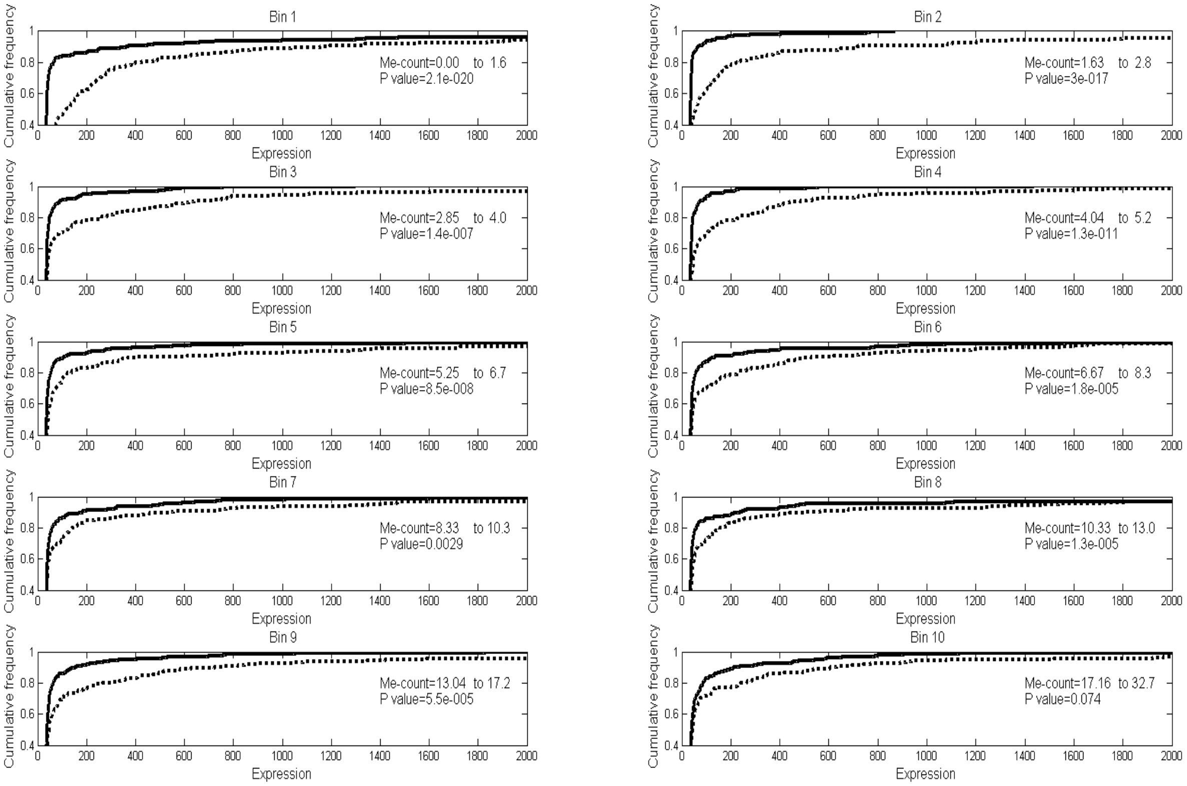

# Figure S10

ES-F

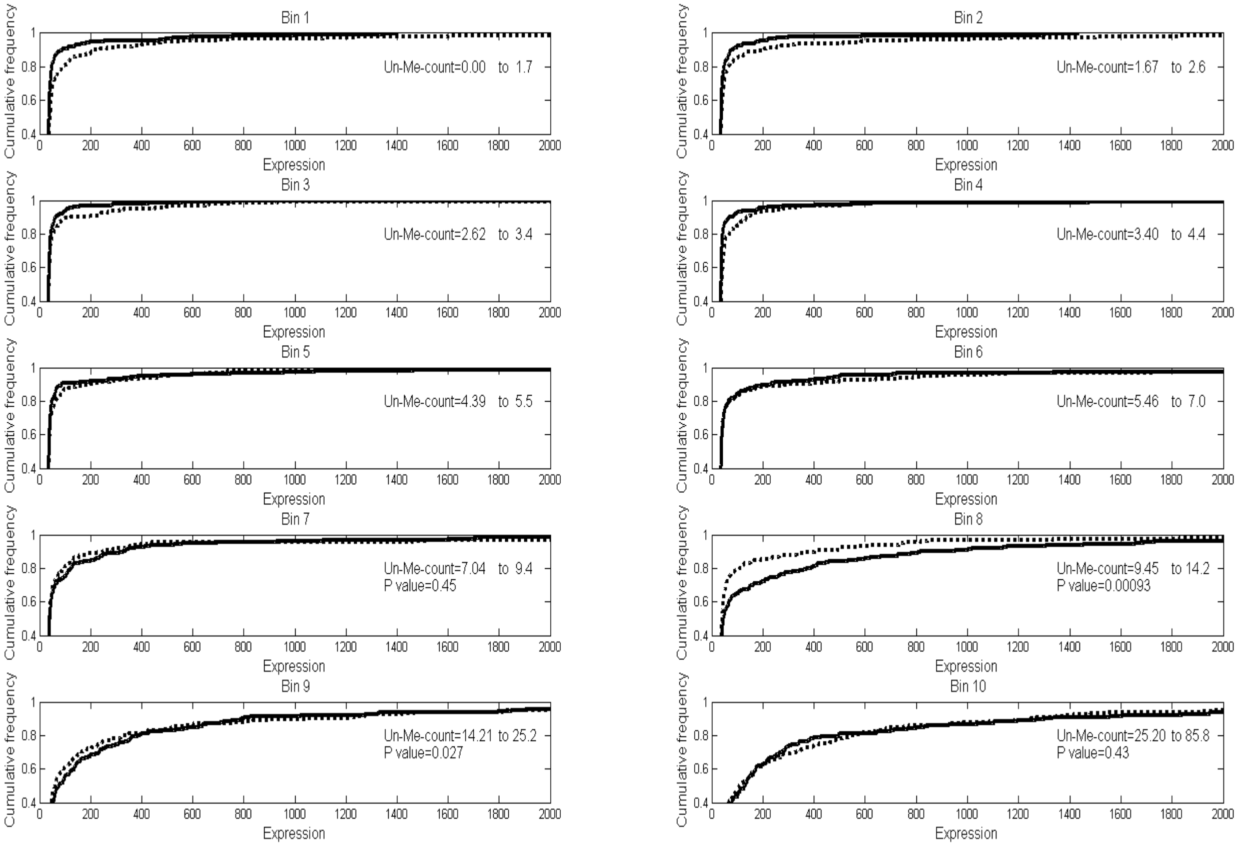

Figure S11

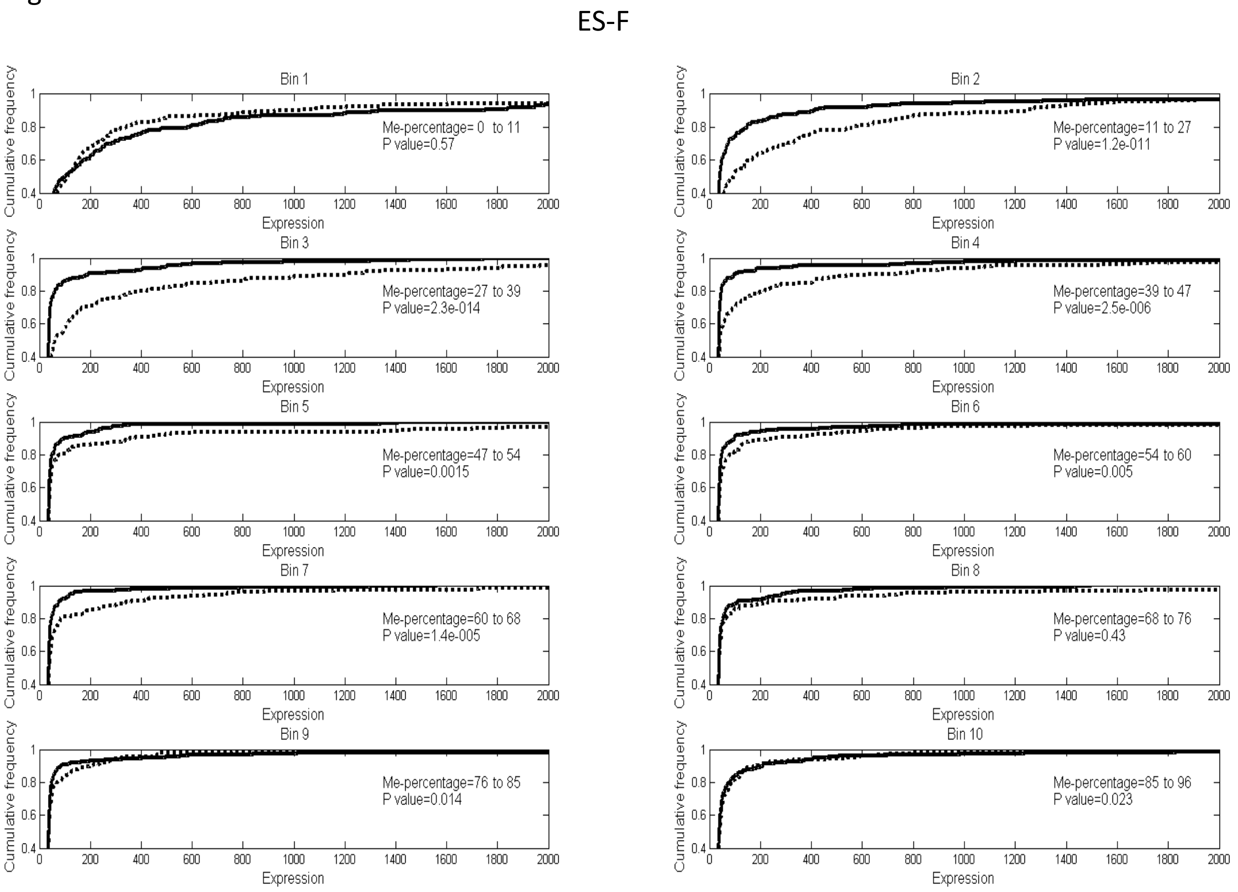
ES-F
Bin 1
Me-percentage= 0 to 11
P value=0.57
Bin 2
Me-percentage=11 to 27
P value=1.2e-011
Bin 3
Me-percentage=27 to 39
P value=2.3e-014
Bin 4
Me-percentage=39 to 47
P value=2.5e-006
Bin 5
Me-percentage=47 to 54
P value=0.0015
Bin 6
Me-percentage=54 to 60
P value=0.005
Bin 7
Me-percentage=60 to 68
P value=1.4e-005
Bin 8
Me-percentage=68 to 76
P value=0.43
Bin 9
Me-percentage=76 to 85
P value=0.014
Bin 10
Me-percentage=85 to 96
P value=0.023
Cumulative frequency
Expression

Figure S12

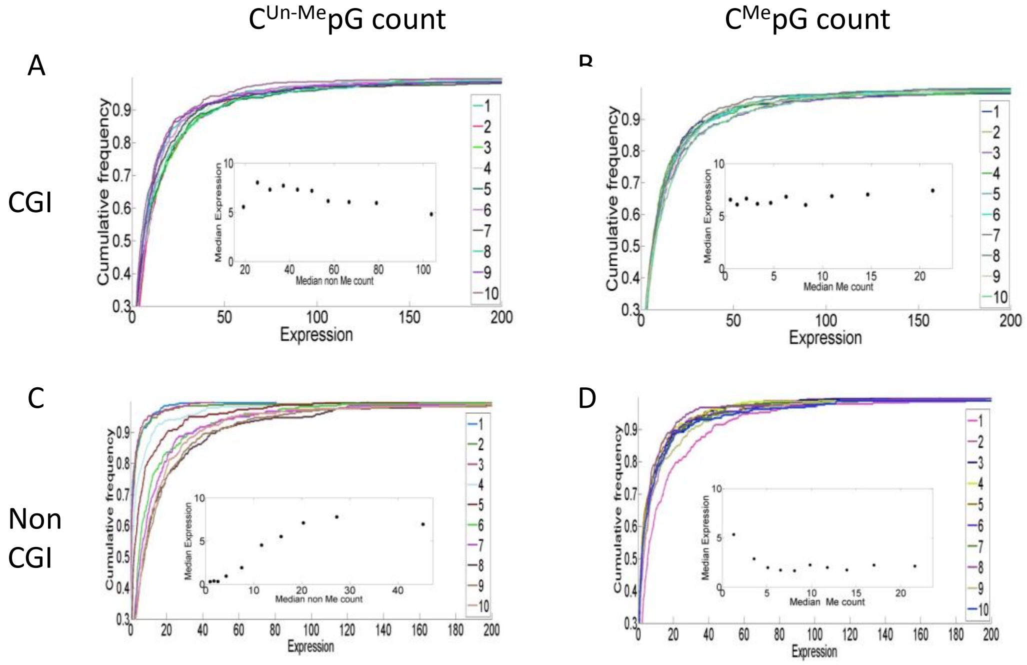

C^Un-Me pG count
C^Me pG count
A
B
CGI
C
D
Non CGI
Cumulative frequency
Expression
Median Expression
Median non Me count
Median Me count

Figure S13

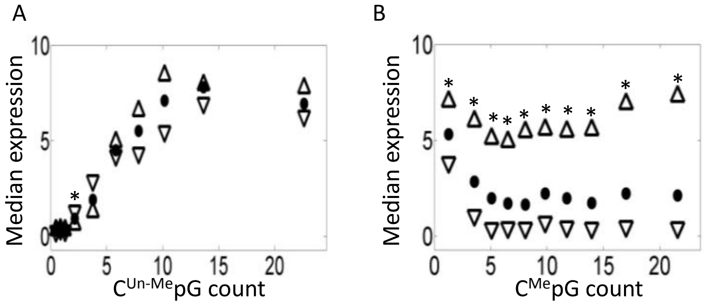


Figure S14

ES-different binning method

$C^{Un\text{-}Me}pG$ count

$C^{Me}pG$ count

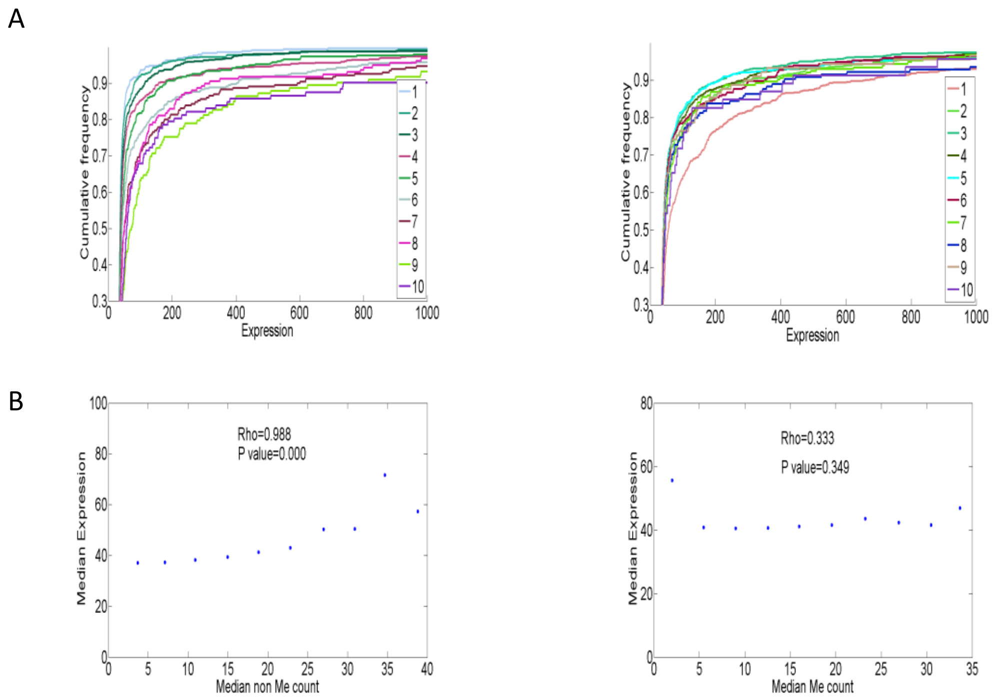

Figure S15

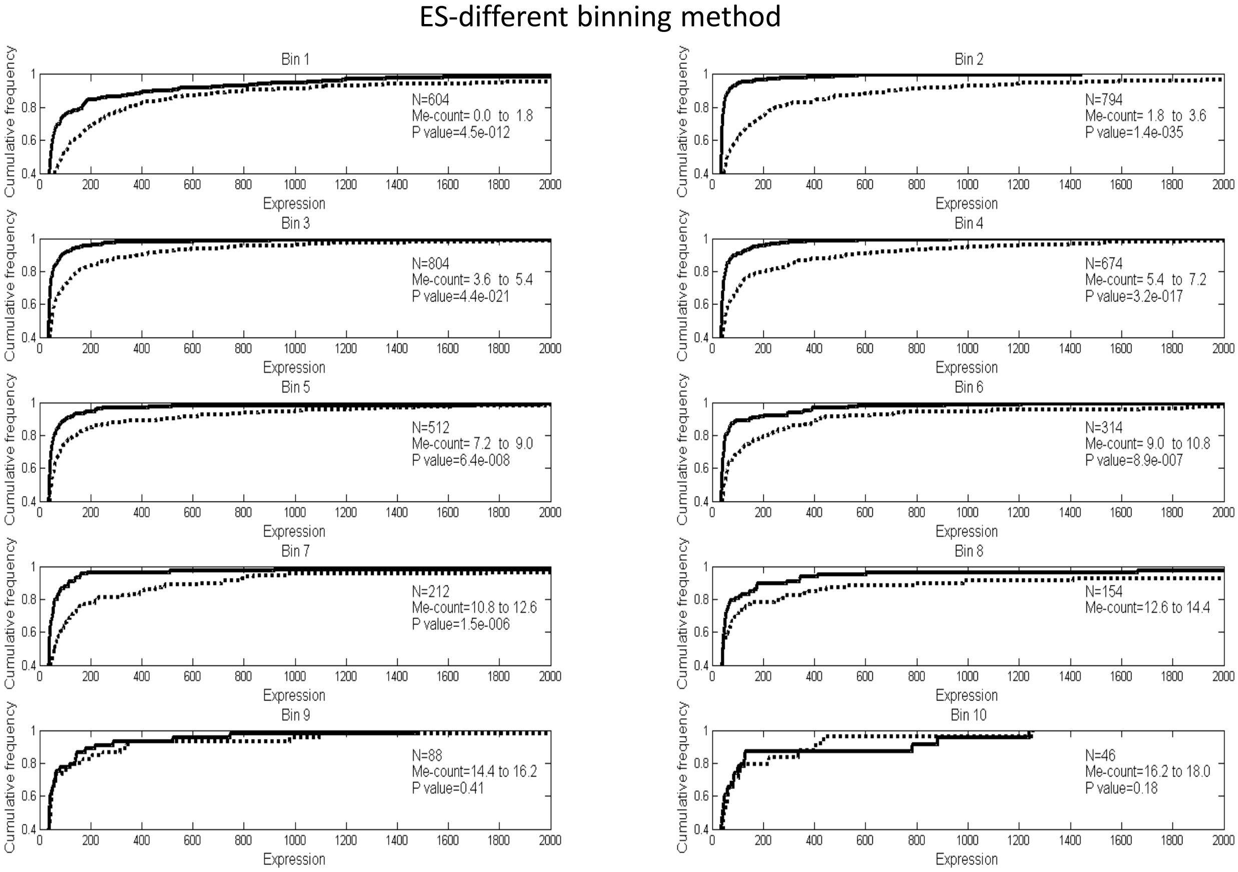
ES-different binning method
Bin 1
N=604
Me-count= 0.0 to 1.8
P value=4.5e-012
Bin 2
N=794
Me-count= 1.8 to 3.6
P value=1.4e-035
Bin 3
N=804
Me-count= 3.6 to 5.4
P value=4.4e-021
Bin 4
N=674
Me-count= 5.4 to 7.2
P value=3.2e-017
Bin 5
N=512
Me-count= 7.2 to 9.0
P value=6.4e-008
Bin 6
N=314
Me-count= 9.0 to 10.8
P value=8.9e-007
Bin 7
N=212
Me-count=10.8 to 12.6
P value=1.5e-006
Bin 8
N=154
Me-count=12.6 to 14.4
Bin 9
N=88
Me-count=14.4 to 16.2
P value=0.41
Bin 10
N=46
Me-count=16.2 to 18.0
P value=0.18
Cumulative frequency
Expression

Figure S16

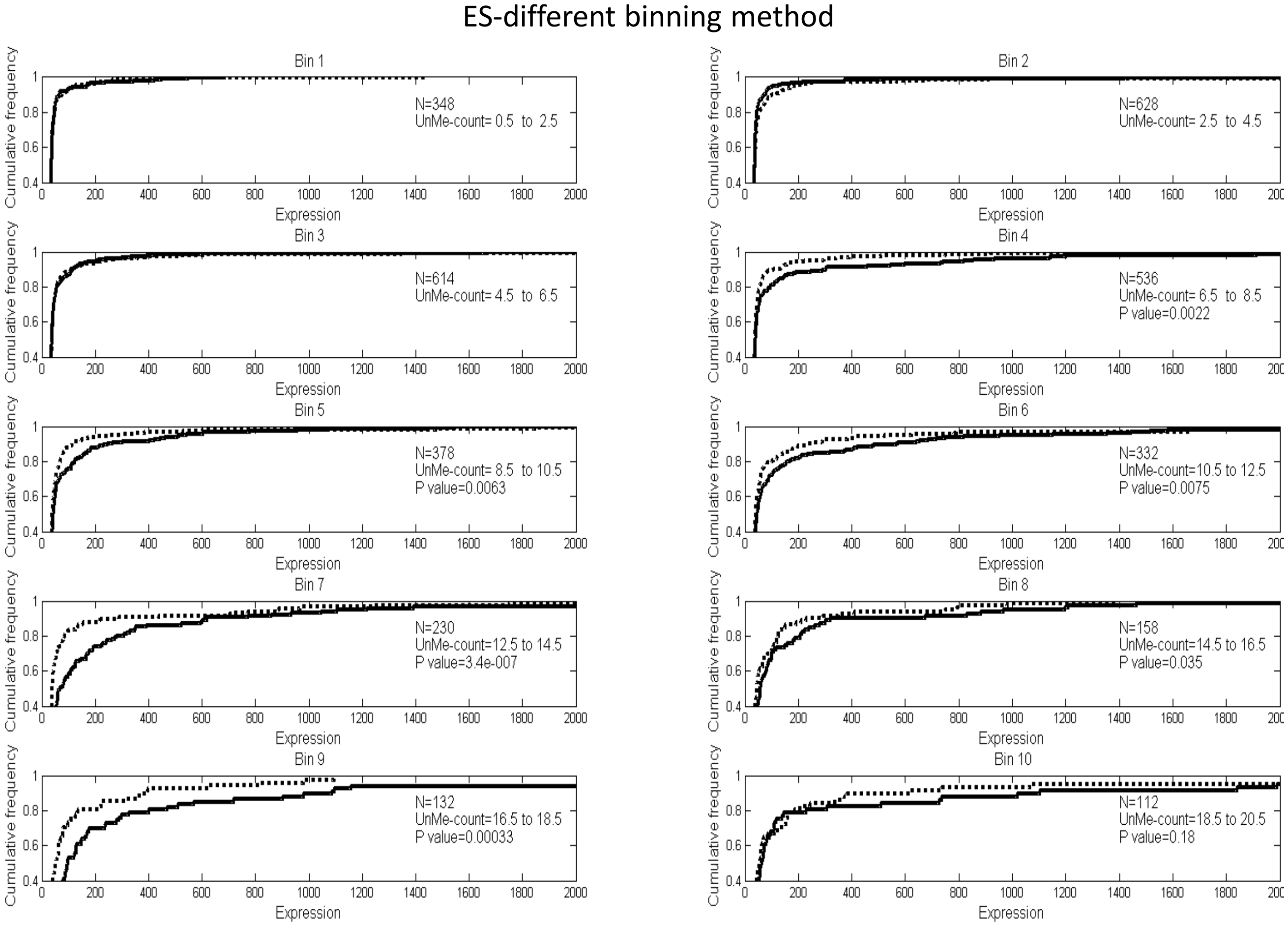
ES-different binning method
Bin 1
N=348
UnMe-count= 0.5 to 2.5
Bin 2
N=628
UnMe-count= 2.5 to 4.5
Bin 3
N=614
UnMe-count= 4.5 to 6.5
Bin 4
N=536
UnMe-count= 6.5 to 8.5
P value=0.0022
Bin 5
N=378
UnMe-count= 8.5 to 10.5
P value=0.0063
Bin 6
N=332
UnMe-count=10.5 to 12.5
P value=0.0075
Bin 7
N=230
UnMe-count=12.5 to 14.5
P value=3.4e-007
Bin 8
N=158
UnMe-count=14.5 to 16.5
P value=0.035
Bin 9
N=132
UnMe-count=16.5 to 18.5
P value=0.00033
Bin 10
N=112
UnMe-count=18.5 to 20.5
P value=0.18
Cumulative frequency
Expression

Figure S17

WT data from Blackledge et al., 2010

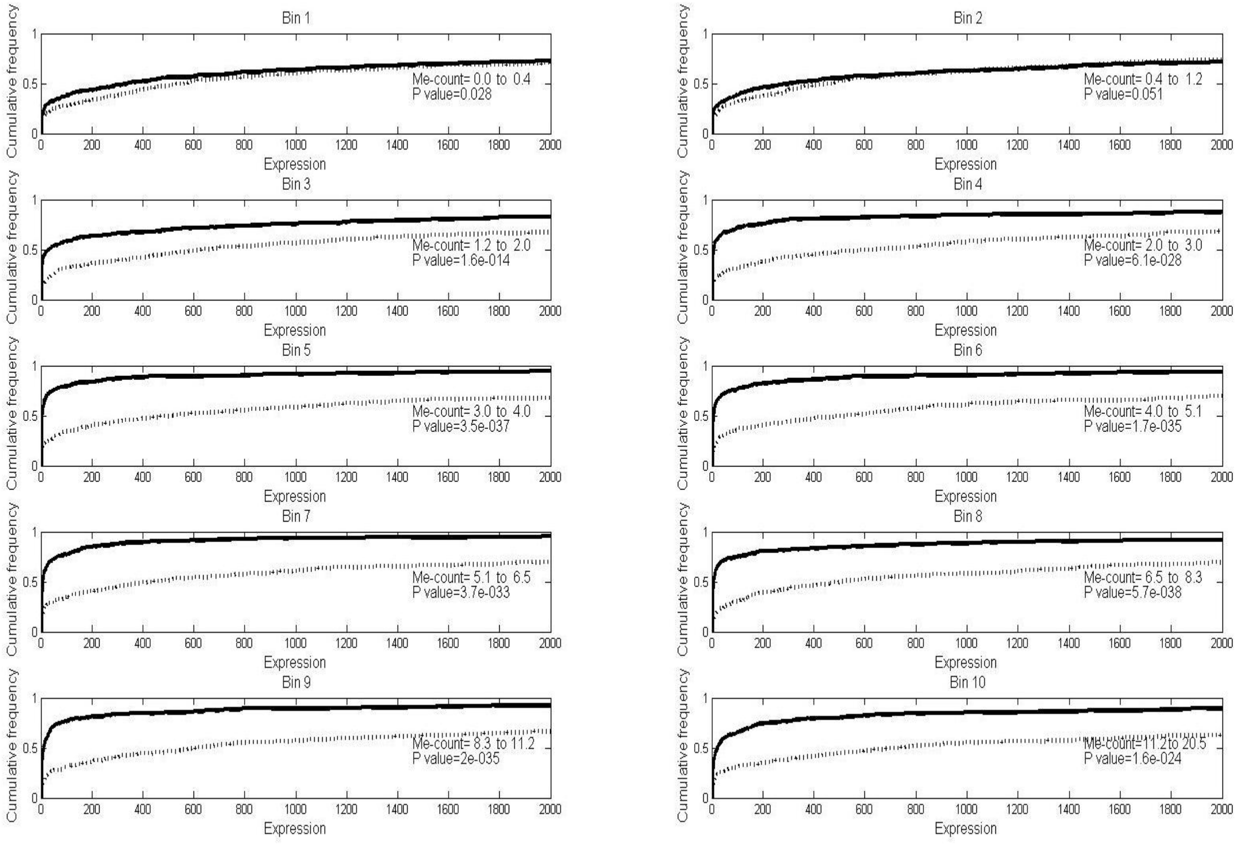

Figure S18

KD2Ma KD data from Blackledge et al., 2010

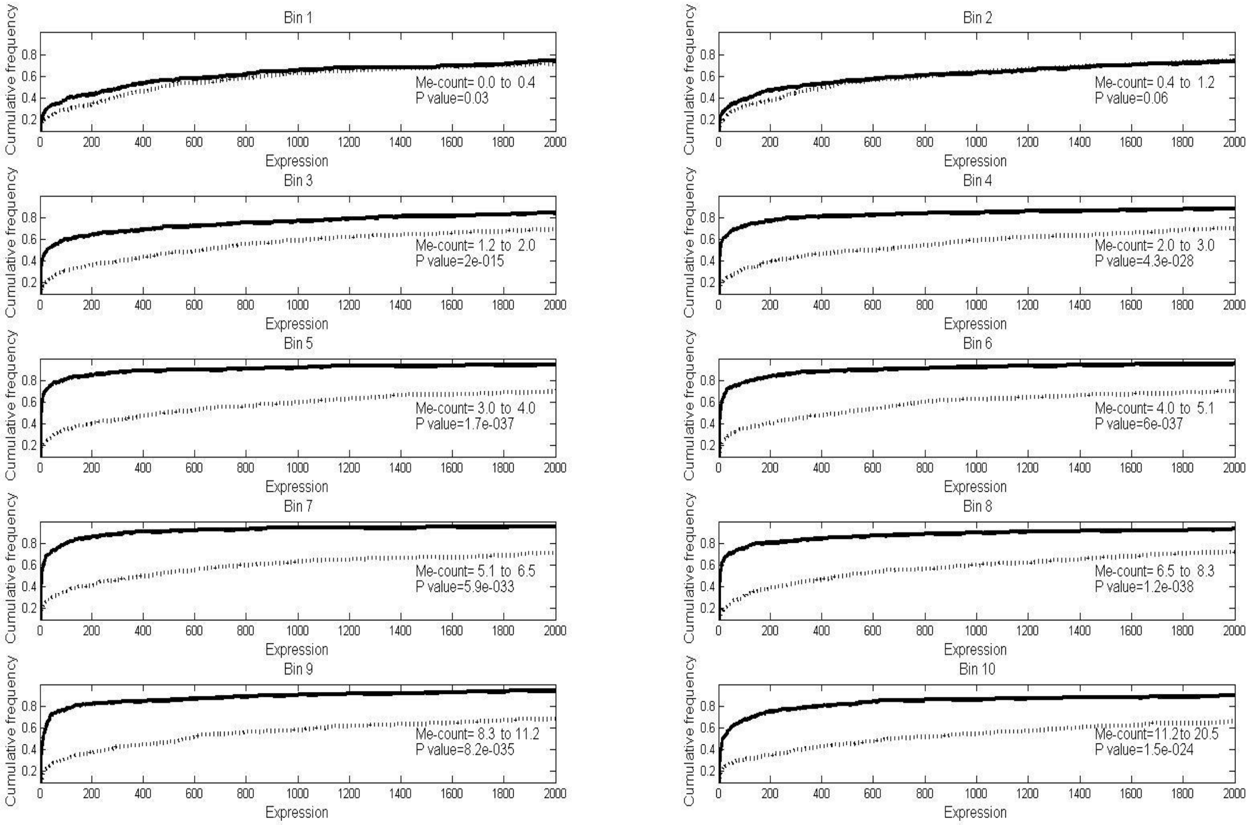

Figure S19

# 20 bins

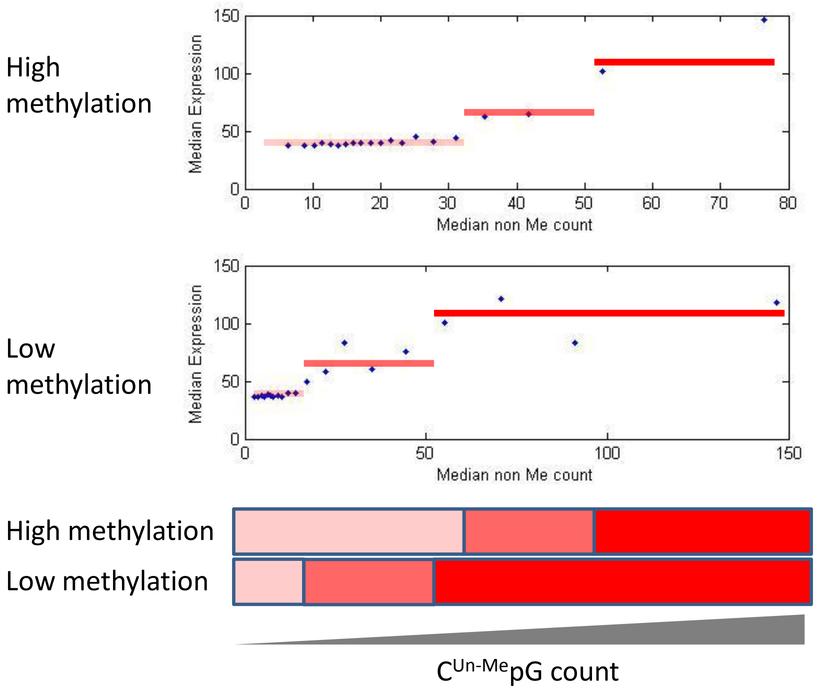

Supplement: Supplementary file 1 — In order to strengthen the argument demonstrated in figure 2 we present the cumulative distributions of all bins separated according to CMepG count, CUn-MepG and CMepG percentage in figures S1, S2 and S3 respectively. The same results were obtained also in primary fibroblast cells (Figures S4-S7) and in ES-F cells (Figures S8-S11). The data was also insensitive to the source of the methylome data since we got essentially the same results using a different methylome data (Figures S12-S13). The results were also insensitive to the binning method since using a different binning method gave essentially the same results (Figures S15-S16) Repeating the experiment using expression data from cells containing either WT levels or KD KDM2a (Blackledge et al., 2010) reveals that KDM2a is not responsible for the higher expression in promoters with many CUn-MepG (Figures S17-S18). Figure S19 is similar to Figure 4 using 20 bins instead of 10 bins. [file 785731.f1.pdf]
